# Exploring spatiotemporal changes in the multi-granularity emotions of people in the city: a case study of Nanchang, China

**DOI:** 10.1007/s43762-021-00030-x

**Published:** 2022-01-04

**Authors:** Xin Xiao, Chaoyang Fang, Hui Lin, Li Liu, Ya Tian, Qinghua He

**Affiliations:** 1 School of Geography and Environment, Jiangxi Normal University, Nanchang, 330022 China; 2 Key Laboratory of Poyang Lake Wetland and Watershed Research, Ministry of Education, Jiangxi Normal University, Nanchang, 330022 China; 3 School of Information Engineering, Jiangxi University of Technology, Nanchang, 330098 China

**Keywords:** Urban emotions, ESTDA, Word shift graph, Social media, User-generated content

## Abstract

In the Internet age, emotions exist in cyberspace and geospatial space, and social media is the mapping from geospatial space to cyberspace. However, most previous studies pay less attention to the multidimensional and spatiotemporal characteristics of emotion. We obtained 211,526 Sina Weibo data with geographic locations and trained an emotion classification model by combining the Bidirectional Encoder Representation from Transformers (BERT) model and a convolutional neural network to calculate the emotional tendency of each Weibo. Then, the topic of the hot spots in Nanchang City was detected through a word shift graph, and the temporal and spatial change characteristics of the Weibo emotions were analyzed at the grid-scale. The results of our research show that Weibo’s overall emotion tendencies are mainly positive. The spatial distribution of the urban emotions is extremely uneven, and the hot spots of a single emotion are mainly distributed around the city. In general, the intensity of the temporal and spatial changes in emotions in the cities is relatively high. Specifically, from day to night, the city exhibits a pattern of high in the east and low in the west. From working days to weekends, the model exhibits a low center and a four-week high. These results reveal the temporal and spatial distribution characteristics of the Weibo emotions in the city and provide auxiliary support for analyzing the happiness of residents in the city and guiding urban management and planning.

## Introduction

The city is not a cold concrete space, and it carries people’s rich emotional experience. The differences in the types and intensity of emotions in different parts of the city and in different public groups can reflect the macroscopic social production and thus the spatial distribution of the social classes in the city. In such diverse urban events as politics (Matalon et al., 2021), economy (Wan et al., 2021), flu viruses (Alamoodi et al., 2021), and natural disasters (Ragini et al., 2018), people’s emotional information and opinions are important factors that reflect the development direction and consequences of the event. The emotional characteristics of people are the product of the social structure and culture of the city. Emotions and space are also fundamentally intertwined (Mody et al., 2009). Therefore, as an important dimension in people’s productivity and life, emotions should be valued by urban planners to ensure that the city’s design is truly people-oriented.

Moreover, driven by the dual development of Internet technology and intelligent mobile devices, every person can be regarded as a sensor in the city (Goodchild, 2007), the restrictions of time and space can be broken, and the Internet can be used to share their views and emotions on social media platforms in the form of text, pictures, videos, and other user-generated content. People leave fine-grained digital traces on social media platforms about when, where, and what to talk about, and who they talk to (Lin, 2014; Lazer et al., 2015), and this information is an appropriate representation of the real emotions of people. Therefore, the large amount of unstructured social media data has become an important direct source of information for the study of people’s emotions in cities (Lin, 2014; Hu et al., 2020; Yu et al., 2021). The mature development of big data mining technology also provides a path for the analysis of the massive data from social media. In recent years, with the rapid improvement of computing power and the continuous optimization of algorithms, machine learning methods have become increasingly accurate in the field of emotion analysis (Yadav & Vishwakarma, 2020). Using machine learning related techniques to mine and understand the content characteristics of social media, analyze the emotions of social media users, and understand the topics on social media in different areas of the city has attracted the interest of many researchers.

It is well known that human emotions are a mixture of the main emotional expressions such as fear, surprise, sadness, anger, and happiness. Are complex and multi-dimensional (Russell, 1980; Mehrabian, 1996), cannot be simply described. Nonetheless, most previous emotion-related studies have explored one-dimensional aspects, such as positive or negative two classifications (Mitchell et al., 2013; Plunz et al., 2019) and positive, negative, or neutral three classifications (Wang et al., 2020). The coarse-grained emotion classification only observes the polarity change of emotion, ignores the diversity of emotion, and can not grasp people’s different emotional tendencies as a whole. On the other hand, multi- spatial-temporal granular analysis of urban people’s emotions is also very important for fine-grained emotion analysis. However, most of the temporal and spatial pattern analyses of the emotions of urban crowds are currently separated (Plunz et al., 2019; Dai & Wang, 2020). Simple mathematical statistics and spatial analysis can only reveal the temporal and spatial patterns of emotions from a single perspective of time or space. The use of spatiotemporal interaction methods to integrate the time and space dimensions can better reflect the dynamic temporal and spatial characteristics of emotions. Therefore, it is necessary to develop a methodological framework to explore the temporal and spatial distributions of multi-granularity emotions. In this context, the main contributions of this study include:

(1) To improve the performance of emotion classification for the Weibo text, this study trained a novel model for Weibo text based on BERT and a convolutional neural network (CNN) and achieved a 76.66% of high accuracy.

(2) This study reveals more comprehensively and truly reflects the emotions expressed by people in Nanchang on social media based on hotspot analysis and topic mining.

(3) This study reveals that Nanchang residents’ emotions on social media have high spatial-temporal flow and low spatial-temporal locking intensity. This study can aid with analyzing the well-being of urban residents and guiding urban development planning.

## Literature review

Emotion is a psychological phenomenon that reflects the relationship between the individual and the environment or event, and has very high complexity and abstraction. When the objective things meet the individual’s expectations, it can cause positive emotions; when the objective things do not meet the individual’s expectations, it will produce negative emotions. For a long time, there has been no unified classification standard for emotion computing. Some scholars believe that emotions are discrete, and they are divided into 4 categories (Yamada et al., 1995), 6 categories (Ekman & Friesen, 1971), 8 categories (Russell, 2005), or 15 categories (Lazarus, 1993). Some scholars believe that emotions are continuous, and use a two-dimensional representation model (Russell, 1980) or three-dimensional representation model (Mehrabian, 1996) to distinguish different emotions.

Generally, researchers mainly use self-reporting, body sensors, and user-generated content (UGC) to collect the emotions of the target group (Huang et al., 2020a). (1) Self-reporting is a traditional and classic method of obtaining people’s emotions. Although traditional methods are greatly impacted in the era of big data, self-reporting methods are still popular and continue to develop and improve. In a recent study, Galvez-Pol provided participants with a map and asked them to color the areas where they had a happy or sad experience (Galvez-Pol et al., 2021). This method of self-reporting allows the subjects to recall their emotions more accurately. Mody developed a mobile application that enables location-based social context awareness and networking through emotional tagging by the user (Mody et al., 2009), which can collect instant feedback from the subject in real-time. However, it must be noted that respondents do not have enough motivation to expose private information, and when dealing with sensitive personal issues, people tend to lie and fabricate data. Therefore, the impact of the inaccuracy of self-reports on the results must be evaluated in the research. (2) In the past, many studies have used body sensors to recognize emotions and have achieved very high accuracies (Maaoui & Pruski, 2010). Kanjo used the smart bracelet to collect data (including heart rate, body temperature, and other indicators) to analyze the emotions of subjects (Kanjo et al., 2018). Engelniederhammer used wearable devices to capture emotions when walking on city streets (Engelniederhammer et al., 2019). Since body sensors can directly obtain physiological signals to recognize emotions (Jerritta et al., 2011), problems such as suppressing emotions or trying to lie can be avoided. However, because of the cost of body sensors and people’s resistance to wearing them, they cannot be used in studies that require large samples. (3) Decentralized social media not only provides the public with a UGC platform on which to express their emotions and views, but also offers a convenient and fast channel for urban researchers to collect data and determine public opinions (Yu et al., 2021; Plunz et al., 2019; Huang et al., 2020b). Text and photos are two important forms of UGC, both of which contain people’s subjective feelings about the objective information of the city. Many studies on big data have shown that a picture is worth a thousand words (Xiao et al., 2020), but in emotion mining tasks, UGC text seems to be easier to interpret (Huang et al., 2020a). Therefore, in this study, emotions were mainly inferred from the text content of Weibos.

Because of the complexity of emotions, accurate measurement of emotion rhythms at the individual level has proven difficult and unconvincing (Golder & Macy, 2011). Therefore, we must use automated technology to mine the emotions of people in cities from a large number of samples. Traditional emotion analysis methods mainly include a sentiment lexicon and deep learning. The method based on a sentiment lexicon is mainly to match the keywords in the text with the lexicon to analyze the emotional tendency. Researchers have built sentiment lexicons suitable for different languages, including Chinese (Wu et al., 2016), English (Baccianella et al., 2010), and French (Abdaoui et al., 2017). Although these studies have achieved good results, the lexicon-based method relies too much on manuals, rules, and complex feature engineering, which limits the application of this method in practical work. Moreover, when using this method researchers usually do not attempt to take the context of the words or the meaning of a text into account, which may lead to difficulties in accurately determining the emotional content of short texts (Mitchell et al., 2013). The emerging deep learning methods have great advantages in the standardized classification and processing of large data sets. Using deep learning to mine emotions is a trend. In particular, with the gradual application of Bidirectional Encoder Representation from Transformers (BERT) and pre-models, the performance and stability of emotion classification have been significantly improved (Sun et al., 2019; Cui et al., 2019).

In summary, the development of big data and deep learning technology has provided a new perspective on research on the emotions of urban people. However, the current research has not made full use of the research results of fine-grained emotion classification in the field of natural language processing, and most researchers still use the positive-negative classification method to determine the emotional tendency. In addition, most analyses of emotions do not pay enough attention to the spatial and temporal characteristics of emotions and ignore the analysis of emotional changes. Emotions are a critical aspect of how people experience places, and the sharing of these emotions represents a powerful social practice (Mody et al., 2009). Therefore, in this study, an extended analysis of the granularity of emotions and the granularity of the temporal and spatial changes was conducted.

## Materials and methods

### Study area and data description

**Fig. 1:**
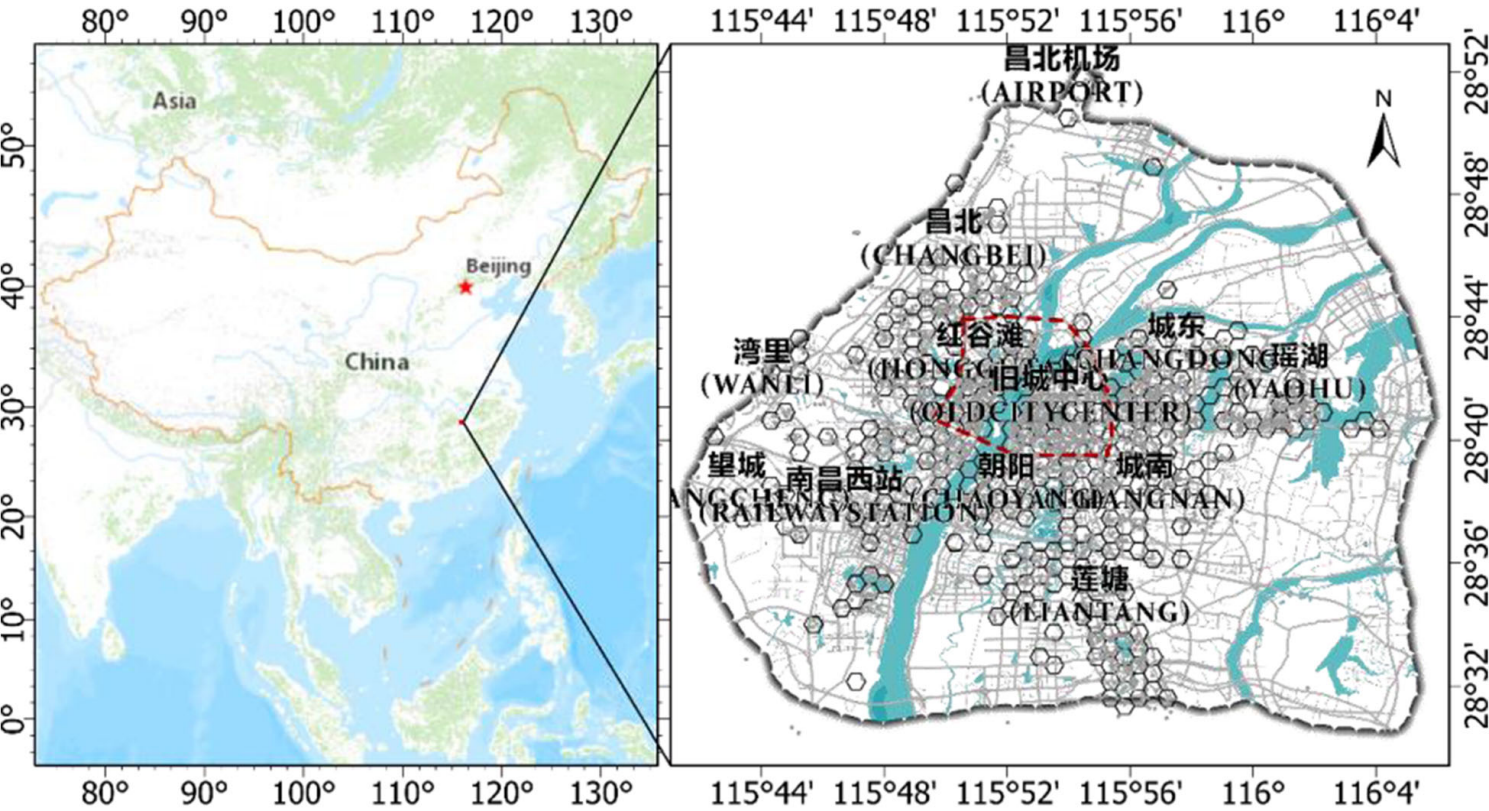
Study area: Nanchang city. The red line indicates the first ring in Nanchang. Each gray point represents a Weibo check-in point

This study was conducted in Nanchang City, the capital of Jiangxi Province, China. Nanchang is the political, economic, cultural, scientific, educational, and transportation center of Jiangxi Province. It is also an important central city in the middle reaches of the Yangtze River. As of 2018, Nanchang contained 6 districts and 3 counties, with a total area of 7402 km^2^. The area of the district is 317.3 km^2^, with a resident population of 6.25 million. We mainly studied the central urban area within the Third Ring express loop highway (Fig. 1). Nanchang’s important business districts, high-level hospitals, and several college towns are contained within this boundary. Compared with other provincial capitals, Nanchang’s historical development has been relatively backward; however, Nanchang City has begun to accelerate its development. For example, many projects such as subway construction and renovation of the old city are underway. Therefore, we hope to determine the characteristics of the emotions of the people in Nanchang during this stage of rapid development.

Sina Weibo (https://weibo.com/), China’s most popular social media application, is widely used, and the number of users is ever-growing. It plays an important role in information dissemination. This platform affects people’s lives in various aspects, including massive information dissemination, faster information discovery, and connection with the world. We developed an automated program to obtain the Weibo data entered by users in the study area. After preprocessing, including deduplication, short text (less than 4 words) deletion, and advertisement deletion, 211,526 pieces of data were retained. Due to the open rules of the Weibo, the data obtained are not all Weibos in Nanchang.

To facilitate the spatial visualization and comparative analysis of the Weibo data in different regions, we gridded the data. Considering that a regular hexagon has the highest spatial tightness and will not be empty during splicing, after comprehensive consideration, we used a regular hexagon to grid the Weibo check-in point data (Fig. 1). If the number of Weibos within the grid is too small, it will affect the credibility of emotion analysis. Therefore, we exclude the grid of fewer than 20 Weibos. The subsequent methods were carried out on the regular hexagonal grid scale.

### Emotion multi-classification based on BERT-CNN

As was previously mentioned, there are currently 4, 6, 8, and 15 classification methods for complex and changeable human emotions in the psychology community, and a recognized standard has not yet been developed. To meet the needs of fine-grained emotional computing and the availability of data, we divided the emotions into 6 categories. Specifically, we used the general Weibo dataset opened from the Ninth China National Conference on Social Media Processing (SMP2020-EWECT). The training set of this dataset contains 29,768 Weibos, and the test dataset contains 5000 Weibos. Each Weibo is marked as one of the following six categories: neutral, happy, angry, sad, fearful, and surprised.

**Fig. 2:**
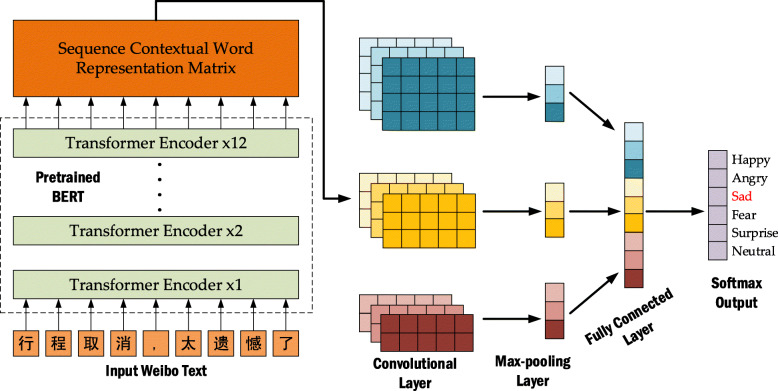
BERT-CNN model structure

To improve the accuracy of the Weibo text emotion analysis, we designed a novel emotion analysis method for Weibo text based on BERT and a convolutional neural network (CNN). The BERT-CNN model was applied to the SMP2020-EWECT dataset. The model structure is shown in Fig. 2. First, the BERT pre-training model (Cui et al., 2019) was used to generate the Weibo text vector through fine-tuning, and then, the CNN was designed to learn high-level abstract features. In other words, we used the embedding extracted from BERT as the conventional embedding layer. In the CNN module, the convolution layer completes the capture of the local important information in the Weibo text features, and the max-pooling layer completes the extraction of the local important features. Then, the final output vector was obtained by stitching through the fully connected layer. Finally, the vector can obtain the probabilities of the different emotion categories through the softmax analyzer.

### Spatiotemporal pattern mining of emotions in the city

Commonly used professional GIS software such as ArcGIS and Geoda provides calculation tools for Moran’I and Getis-Ord Gi* statistics (hot spot analysis) (Getis & Ord, 1992) to explore the spatial pattern of data. Their formulas can be found in the user guide of the software (Mitchell, n.d.). However, individual emotion will change with the change of location and time, there must be temporal and spatial differences in emotion in urban space. To mine the temporal and spatial distribution characteristics of emotion, the exploratory spatiotemporal data analysis (ESTDA) framework (Rey & Janikas, 2006) is introduced.

The Moran’s I index can measure and analyze the local indicators to determine the spatial associations between the different emotions in urban areas, while the LISA time path analysis in ESTDA studies the dynamic migration law of the local autocorrelation in the Moran scatter diagram from the perspective of time evolution by introducing the time dimension. The relative length of the LISA time path is calculated as follows:

$$ {T}_i=\frac{n\times \sum {\sum}_{t=1}^{T-1}d\left({L}_{i,t},{L}_{i,t+1}\right)}{\sum \limits_{i=1}^n\sum \limits_{t=1}^{T-1}d\left({L}_{i,t},{L}_{i,t+1}\right)} $$

where n is the number of hexagonal grids; T is the time interval; *L*_*i*, *t*_ is the LISA coordinate of hexagonal grid *i* at time t; and *d*(*L*_*i*, *t*_, *L*_*i*, *t* + 1_) is the moving distance of the hexagonal grid from time t to *t* + 1. The larger the value of *T*_*i*_ is, the less stable the migration path of the Moran’s I scatter is over time, that is, the regional emotion has a more dynamic local spatial dependence and local spatial structure.

The LISA time path describes the geometric law of the migration trajectory of each element on the Moran’s I scatter diagram, and the spatiotemporal transition describes how the spatial relationship between the hexagonal grid and its adjacent grid changes with time. The evolution of the Local Moran’s I scatter gram between different local types can be characterized by the transition probability matrix and the spatiotemporal transition. There are four types of spatiotemporal transitions: I, II, III, and IV (Rey, 2001). The Type I transition means that hexagonal grid i experienced no transition between morphologies during the study period. The Type II transition means that the hexagonal grid itself experienced a transition, but the neighboring grid did not experience a transition, and it is divided into HH → LH, HL → LL, LH → HH, and LL → HL. The Type III transition indicates that the hexagonal grid itself has not changed, but its adjacent grids experienced transitions, including HH → HL, HL → HH, LH → LL, and LL → LH. The Type IV transition means that both the hexagonal grid itself and the neighboring grid experienced transitions, and the transition direction of the city itself and the neighboring grid is the same. It is defined as type IV_(a)_, including HH → LL and LL → HH. The transition directions of the hexagonal grid itself and the neighboring grids are inconsistent, which is defined as type IV_(b)_, including HL → LH and LH → HL. The spatiotemporal flow (F) and spatiotemporal cohesion (C) can be used to characterize the path dependence and lock-in characteristics of the spatial pattern of a place. The equations are as follows:

$$ F=\frac{S_{\mathrm{II}}+{S}_{\mathrm{II}\mathrm{I}}}{m} $$

$$ C=\frac{{\mathrm{S}}_{\mathrm{I}}+{\mathrm{S}}_{\mathrm{I}\mathrm{V}\left(\mathrm{a}\right)}}{\mathrm{m}} $$

where *S*_II_, *S*_III_, and S_IV(a)_ are the number of the spatiotemporal transitions of II, III, and IV_(a)_, respectively, and m is the total number of hexagonal grids.

### Interpreting emotional topics based on word shift graphs

To better capture the fine-grained differences in the Weibo topics between different emotions, we used the word shift graph visual analysis method (Gallagher et al., 2021). Word shift graphs can identify which words most account for the between-text variations, and they can also explain how each word contributes. The simplest word shift graph is to use the proportion of words in different texts to determine the position of the words. Proportion shifts are easy to interpret, but they are simplistic and have a difficult time identifying interesting differences between two texts (Gallagher et al., 2021). Therefore, we measured the Shannon entropy of the Weibo texts’ word distribution in different emotions to understand how the emotional change may have affected the information content of the language used in Weibo.

The entropy is a measure of the unpredictability of the topic in a group in Weibo. The Shannon entropy (Shannon, 1948) is calculated as follows:

$$ H(P)={\sum}_{\uptau \in \mathcal{T}}{p}_{\uptau}{\log}_2\frac{1}{p_{\uptau}} $$

Where *p*_τ_ is the probability of word τ in all words ($\mathcal{T}$) in this group of Weibos.

The larger the entropy is, the richer the words used and the more unpredictable the topics in this group of Weibos are. In contrast, the smaller the entropy is, the more concentrated the words and the single topic in this group of Weibos are. The contribution of each word in the Shannon enterprise shift is calculated as follows:

$$ \updelta {H}_{\uptau}={p}_{\uptau}^{(2)}{\log}_2\frac{1}{p_{\uptau}^{(2)}}-{p}_{\uptau}^{(1)}{\log}_2\frac{1}{p_{\uptau}^{(1)}} $$

Where ${p}_{\uptau}^{(n)}$ is the probability of word t in all words (T) of the Weibo in group n.

## Results

### Emotion classification model training experiment

We used accuracy to evaluate the performance of trained emotion classification models, and calculated the precision score (P), the recall score (R), and the F1-score (F) of each label to get more insight about the effectiveness. The calculation formula is as follows:

$$ \mathrm{Accuracy}=\frac{\mathrm{tp}+\mathrm{tn}}{\mathrm{tp}+\mathrm{fp}+\mathrm{tn}+\mathrm{fn}} $$

$$ \mathrm{Precision}\left(\mathrm{P}\right)=\frac{\mathrm{tp}}{\mathrm{tp}+\mathrm{fp}} $$

$$ \mathrm{Recall}\left(\mathrm{R}\right)=\frac{\mathrm{tp}}{\mathrm{tp}+\mathrm{fn}} $$

$$ \mathrm{F}1-\mathrm{score}\left(\mathrm{F}\right)=\frac{2P\ast R}{\mathrm{P}+\mathrm{R}} $$

Where tp is true positive; fp is the number false positive classifications; tn is the number of true negative classifications and fn is the number of false negative classifications.

The emotion classification model achieves a 76.66% accuracy upon testing set. Confusion matrix on the testing set along with precision, recall and F1-scores metrics for each emotion category are summarized in Table 1. It can be seen from the data in Table 1 that the trained model gave the best performance on the “neutral” label followed by the “anger” and “happy” labels. The obtained F1-score of these labels was above 75%. The worst performance was obtained on the “surprise” label. Despite our model cannot completely accurately identify the emotion type of Weibo text, its performance still reaches a relatively high level for the complexity of Chinese.

**Table 1:** Performance analysis of trained emotion classification model

	Confusion matrix						Evaluation metrics		
	Happy	Angry	Sad	Fear	Surprise	Neutral	P	R	F
Happy	0.813	0.024	0.050	0.013	0.028	0.067	0.729	0.813	0.769
Angry	0.040	0.791	0.114	0.011	0.029	0.016	0.851	0.791	0.820
Sad	0.109	0.131	0.690	0.029	0.013	0.028	0.673	0.690	0.681
Fear	0.071	0.038	0.110	0.710	0.033	0.038	0.618	0.710	0.661
Surprise	0.150	0.112	0.048	0.072	0.586	0.032	0.648	0.586	0.615
Neutral	0.080	0.017	0.032	0.010	0.029	0.831	0.857	0.831	0.844

### Results of the emotion classification

The trained emotion classification model was used to judge the emotional tendencies of all of the Weibo samples. Table 2 shows that non-neutral interests account for 81.49%. Some studies have found that people like to post emotional content on the Weibo platform through offline interviews (Vermeulen et al., 2018). However, previous research has also shown that people seem to prefer to post negative emotions on Twitter, and our research shows that people in Nanchang have similar probabilities of posting positive emotions and negative emotions on Weibo. In detail, people seem to prefer to post content that implies happy (28.82%), angry (16.61%), and sad (24.09%) emotions on Weibo, and fewer people express their own fear and surprise emotions.

**Table 2:** Results of emotion classification

	Happy	Angry	Sad	Fear	Surprise	Neutral
count of Weibo	65,766	37,876	54,984	1611	9073	42,216
proportion	28.84%	16.61%	24.09%	0.71%	3.98%	18.51%

### Spatial distribution and topic of emotions

**Fig. 3:**
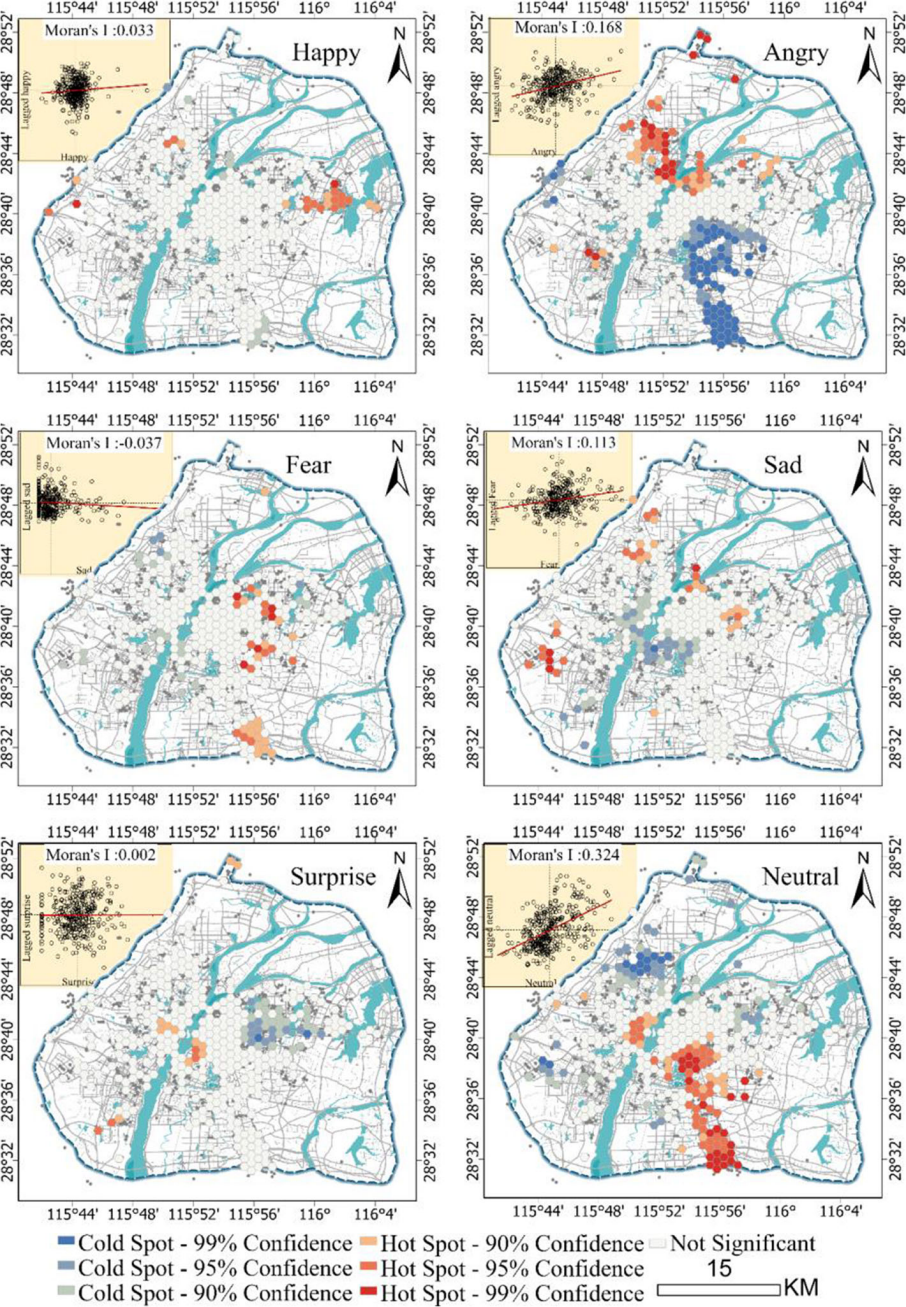
Spatial clustering patterns of the six emotions

Figure 3 shows the spatial distribution characteristics of the six types of emotions (happy, angry, fearful, sad, surprised, and neutral) in Nanchang. Angry and neutral have the highest global Moran’s I index values, so the distributions of these two types of emotions exhibit a strong spatial correlation. The global Moran’s I index values of happy and fearful are slightly lower than those of the previous two, indicating a lower degree of spatial correlation. The sad global Moran’s I index value is slightly less than 0, indicating that this emotion exhibits a certain spatial difference. In addition, the surprised emotion is almost equal to 0, so this emotion is almost randomly distributed in the city.

The emotion map obtained by hot spot analysis combines subjective and intangible emotion with objective and tangible space, which can intuitively and clearly show the distribution characteristics of emotion in different spatial environments in Nanchang. Combined with the word shift graph, we can find the topics talked about by people living in Nanchang.

#### Happy

**Fig. 4:**
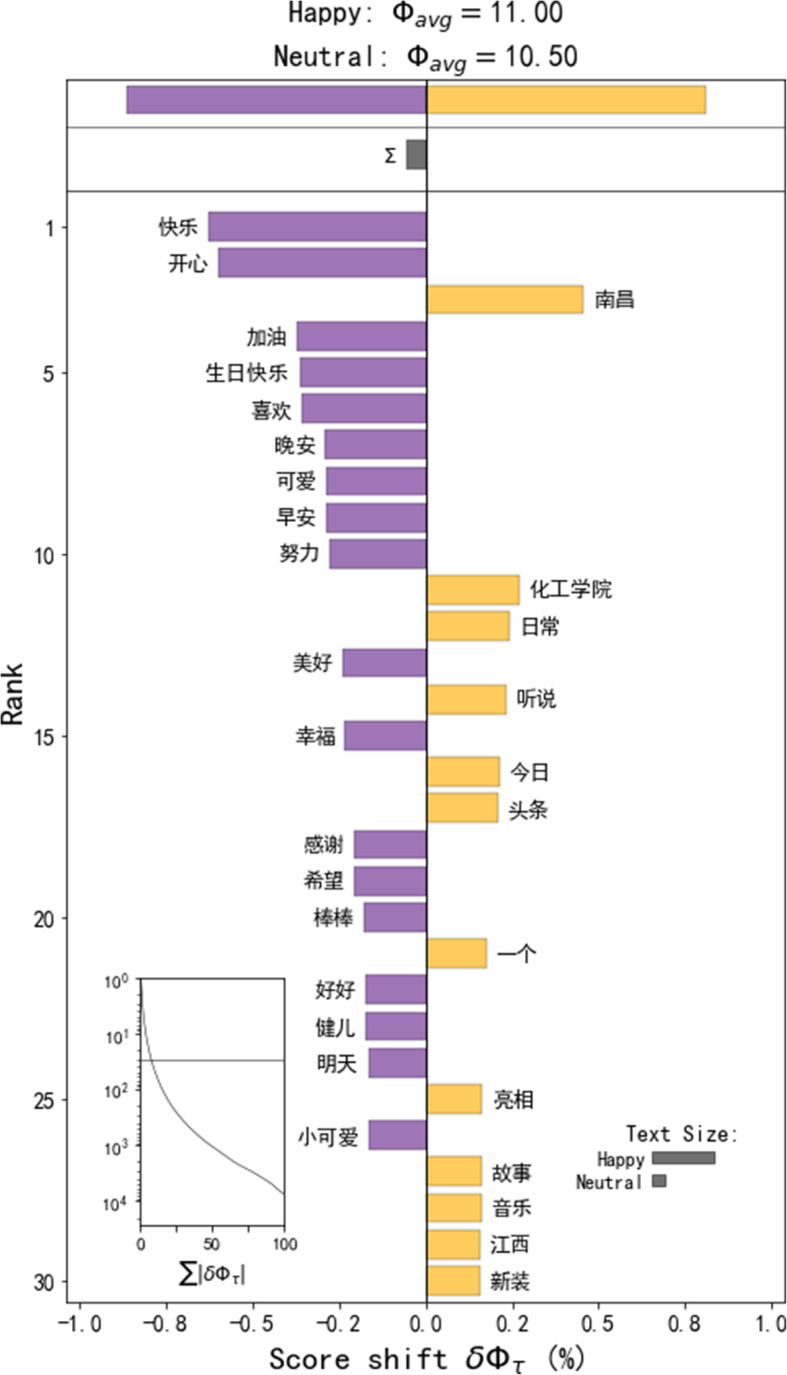
Word shift graphs of the Weibos in the happy emotion hot spots

The happy emotion type in Nanchang is relatively concentrated, forming a large-scale hot spot cluster in Yaohu University Town. Changbei University Town in the northern part of Nanchang and Wanli University Town in the western part of Nanchang has a small range of happy emotion hot spots. In addition, by observing the word shift graph (Fig. 4), we found that there are many positive words in the Weibo that imply happiness, and most of them are related to campus life. This shows that university towns seem to be more likely to become hot spots for happy emotions, and the people here prefer to share positive emotions. The information entropy of Weibo with happy emotions is greater than that of Weibo with neutral emotions, indicating that people discuss more happy topics in these places.

#### Angry

**Fig. 5:**
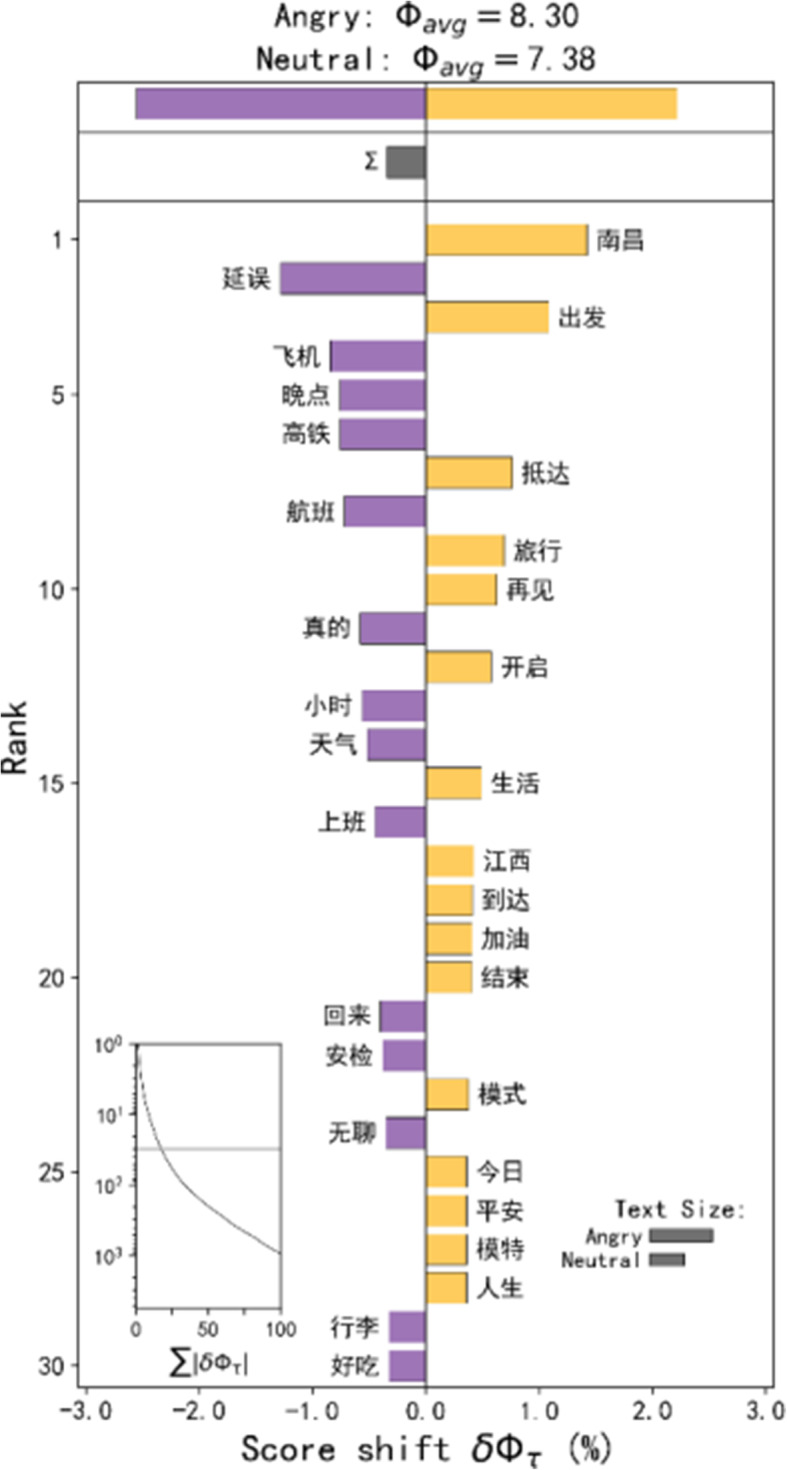
Word shift graphs of the Weibos in the angry emotion hot spots

The hot spots of angry emotions are mainly distributed in the northern, northernmost, and southwestern regions of Nanchang City. Separately, there are complex urban infrastructure and functional communities in the northern area, while the northernmost hot spot can be directly judged to be Changbei International Airport, and the hot spot in the southwest is the high-speed rail station. The word shift graph (Fig. 5) shows that the angry emotion topics in these areas are more diverse; however, it can be reasonably assumed that the anger in these places is related to flight or high-speed rail delays and security checks.

#### Sad

**Fig. 6:**
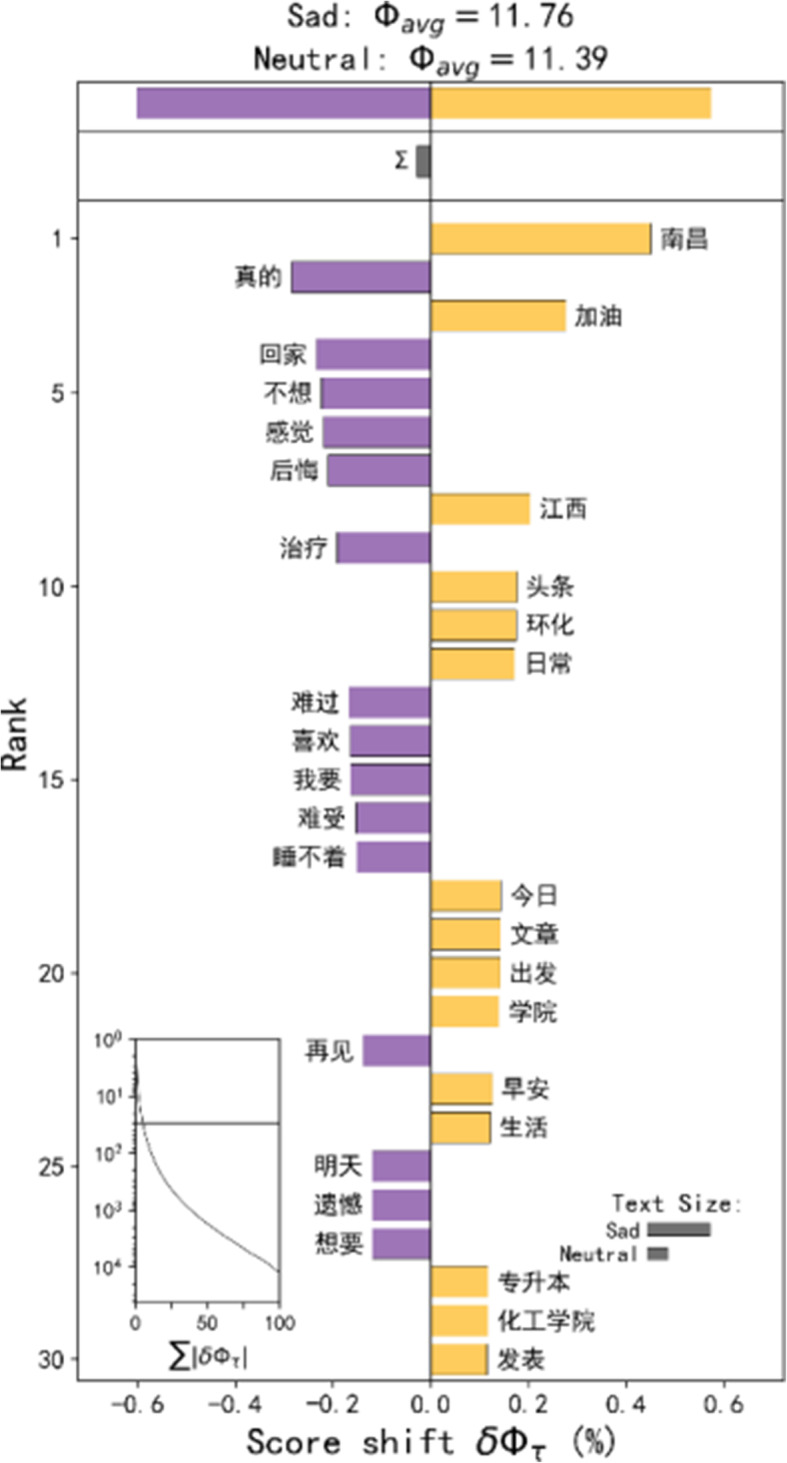
Word shift graphs of the Weibos in the sad emotion hot spots

The sadness seems to be relatively scattered in urban geographical space, and there are hot spots in hospitals, schools, transportation stations, residential areas, and industrial parks. As one of the important negative emotions, people are sad for many reasons. However, on the word shift graphs (Fig. 6), the most prominent comparison of neutral emotions is the words related to medical and health.

#### Fear and surprise

**Fig. 7:**
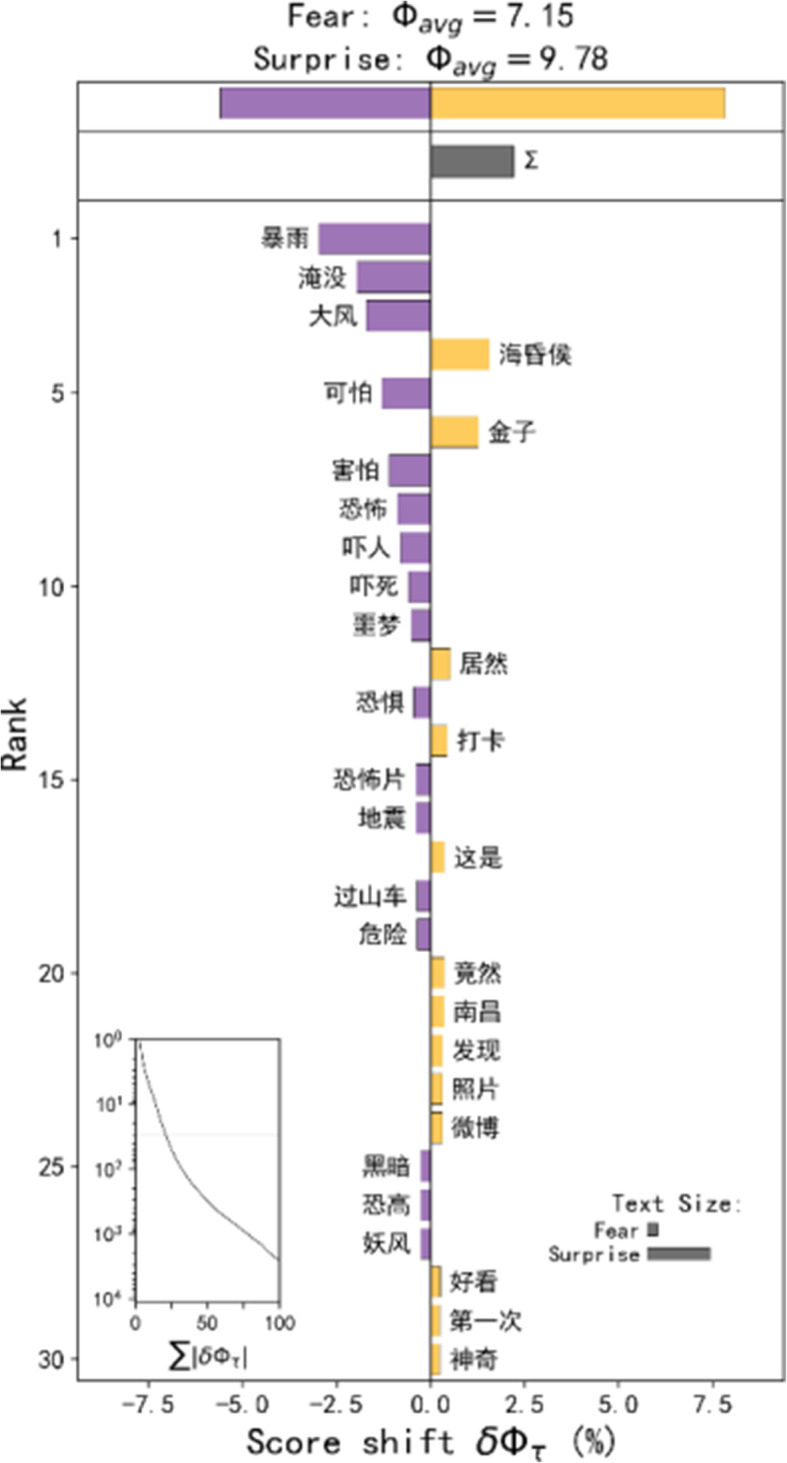
Word shift graphs of the Weibos in the fear and surprise emotion hot spots

Compared with the three emotions assessed above, the emotions of fear and surprise account for a smaller proportion, and their spatial distribution is also relatively discrete. Therefore, we compared all of the Weibo related to these two emotions together. Based on the word shift graphs (Fig. 7), we found that the hot topics of Weibo that are related to fear are mainly related to the weather (high winds and heavy rains). People are surprised at the Hai-Hun-Hou tomb of the Han Dynasty just excavated in Nanchang.

### Analysis of the dynamic spatiotemporal characteristics of emotions

**Fig. 8:**
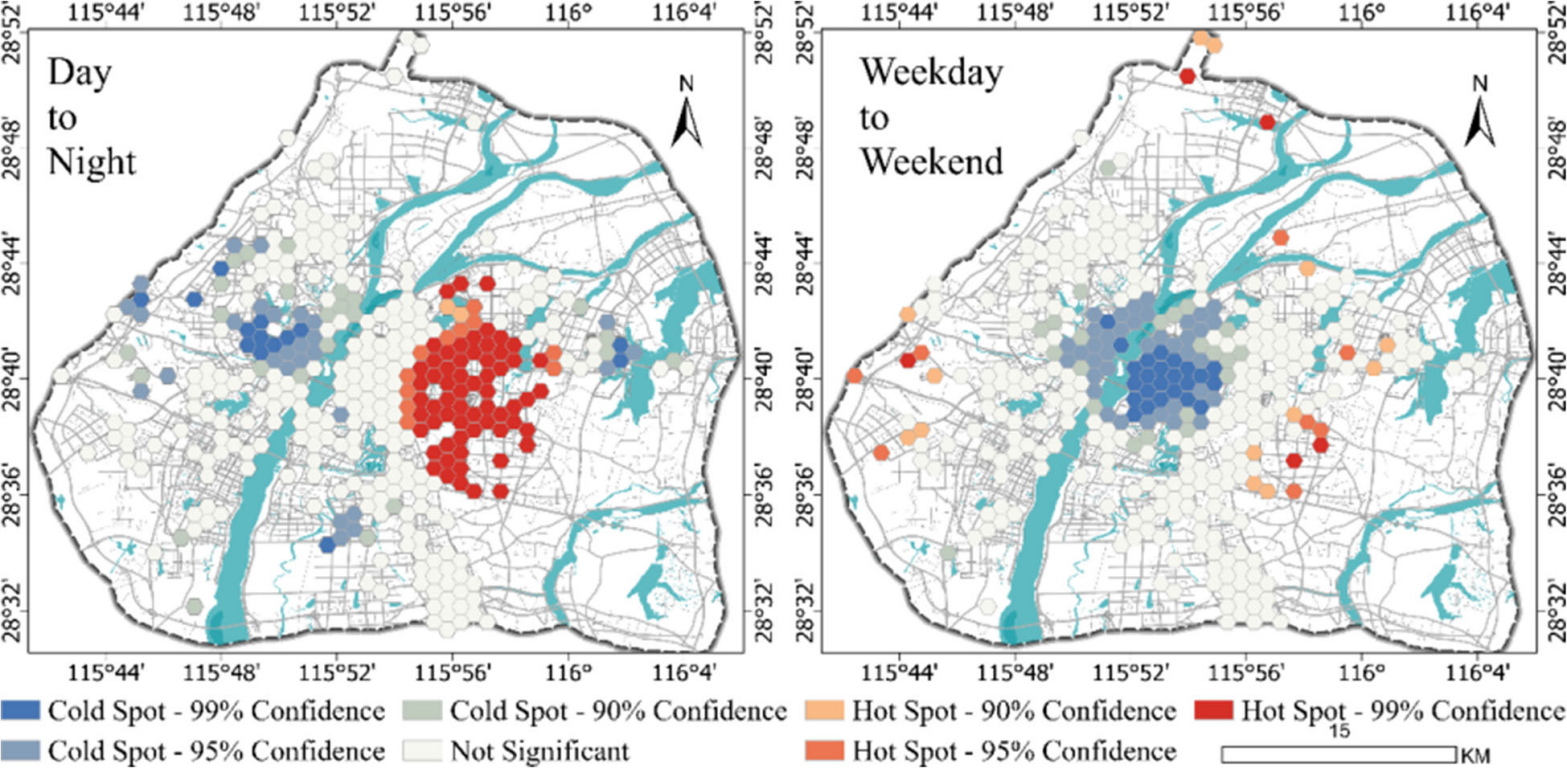
Spatial distribution of hot spots (cold spots) of emotional volatility. The left figure shows the hot spots of the relative length of the time path of emotions from day to night, and the right figure shows the hot spots of the relative length of the time path of emotions from weekday to weekend

The relative length of the LISA time path can reveal the local spatial dependence of the emotions and the stability of the spatial structure from the perspectives of time and space. First, we simulated the specific positions of the six emotional Moran scatter plots in different places in Nanchang during the day and night and on working days and weekends, and then, we calculated the relative lengths of the LISA time paths in different places in Nanchang (Appendix 1 and 2). To further analyze the dynamic characteristics of the emotional spatial structure in Nanchang, the sum of the time path lengths of the six emotions was calculated. Then, ArcGIS was used to analyze the hot and cold areas of the sum of the time paths of the six emotions (Fig. 8).

Figure 8 shows that from day to night, the relative length was generally low in the west and high in the east. This means that people’s emotions in the eastern region have a strong dynamic local spatial structure, and people’s emotions in the western region of the city have a more stable local spatial structure. However, from weekdays to weekends, the relative length was generally low in the center with scattered high values in the surrounding areas. This shows that the local spatial pattern of people’s emotions in the central area of the city exhibited little change, and the spatial structure was relatively stable. People’s emotional local spatial pattern was more unstable and the spatial structure was more dynamic in areas far from the urban center.

Furthermore, the transfer probability matrix and spatiotemporal transition proposed by Rey were used to describe the transfer characteristics and evolution of the local spatial correlation types of the six emotions in Nanchang. (Table 3 and Table 4).

**Table 3:** The spatiotemporal transition of the LISA for the six emotions from day to night

Emotion	I	II	III	IV(a)	IV(b)	SF	SC
Angry	0.60	0.29	0.08	0.01	0.01	0.38	0.61
Happy	0.41	0.23	0.22	0.07	0.07	0.45	0.48
Sad	0.41	0.31	0.19	0.04	0.06	0.50	0.44
Fear	0.42	0.15	0.35	0.05	0.04	0.50	0.47
Surprise	0.43	0.24	0.20	0.06	0.07	0.43	0.49
Neutral	0.64	0.23	0.10	0.01	0.01	0.33	0.66

**Table 4:** The spatiotemporal transition of the LISA for the six emotions from weekday to weekend

Emotion	I	II	III	IV(a)	IV(b)	SF	SC
Angry	0.51	0.24	0.16	0.07	0.03	0.40	0.57
Happy	0.34	0.22	0.28	0.07	0.09	0.50	0.41
Sad	0.44	0.23	0.21	0.05	0.08	0.43	0.49
Fear	0.50	0.14	0.30	0.01	0.05	0.44	0.51
Surprise	0.42	0.25	0.19	0.04	0.10	0.44	0.47
Neutral	0.66	0.23	0.09	0.02	0.01	0.32	0.68

Overall, the distribution of the emotions in Nanchang exhibited strong transfer activity, that is, the spatial pattern of the emotions is characterized by a low path dependence and negligible locking. Most regions have a significant spatiotemporal transition, the spatial cohesion of the emotion distribution is low, the spatial pattern is turbulent, and each region changes its current emotional distribution with time.

Specifically, by comparing the spatiotemporal flow and spatiotemporal cohesion index, the path dependence and locking characteristics of anger are the most obvious for both day to night and weekday to weekend. From day to night, the spatial patterns of sadness and fear are more unstable, and the spatial transfer of these two emotions has higher activity in a day. From weekdays to weekends, the spatial pattern of happiness, a positive emotion, is the most unstable, the spatial cohesion is the weakest, and the hot spots exhibit a significant spatiotemporal transition.

## Discussion

### The spatiotemporal distribution of emotions is heterogeneous

In this study, a BERT-CNN emotion classification model was trained, which combines BERT based on pre-training with a convolutional neural network to give full play to their respective advantages. Compared with the traditional research on dichotomous emotion classification, in this study, six common emotions, which have a higher emotion classification granularity, were studied. In addition, based on the ESTDA model, in this study, the dynamic characteristics of the emotional spatiotemporal characteristics from day to night and from working day to the weekend were analyzed, which overcomes the problem that traditional ESDA only considers space and ignores the time dimension, thus realizing the benign coupling of the temporal and spatial characteristics.

The main findings of this study are as follows. (1) There are differences in the emotions among the people in cities. Among them, the proportion of happiness is the highest. This conclusion is similar to that of a previous study (Huang et al., 2020b). People seem to prefer to share positive emotions in the online environment. (2) There are differences in the emotions and topics in different places in the city. This difference is obvious, and we also found that emotions are richer in the city’s center, so most of the hot spots of the various emotions are distributed around the city instead of in the center. (3) There are also significant differences in the temporal and spatial changes in people’s emotions in cities. Overall, the spatiotemporal flow of emotions is high, and the spatiotemporal locking intensity is low. Specifically, from day to night, the city exhibits a pattern that is high in the east and low in the west. From working days to weekends, the mode is characterized by a low center and a high surrounding.

### Emotional spatiotemporal analysis promotes urban dynamic evaluation

More importantly, this study once again demonstrates that in the Internet age, emotions exist in cyberspace and geospatial space, and social media is the mapping from geospatial space to cyberspace. The subjective perception of the emotions of a city can be a valuable source of information for city planners and local administrations (Pánek et al., 2017). Therefore, city managers should pay attention to the public’s emotional expression on social media, establish effective and smooth channels of expression, and guide the public to express their emotional demands on the Internet in a correct way.

on one hand, emotion analysis and emotion calculation methods can provide effective tools for objective evaluation and monitoring of psychological disorders, especially depression (Zucco et al., 2017; Sharma & Sharma, 2021). Especially affected by covid-19, blockade resulted in higher depression and suicidal tendency. The time series, spatial differentiation and topic analysis of emotion are helpful to reveal the temporal and spatial characteristics and influence mechanism of mental diseases, so as to provide scientific decision-making basis for the prevention and control of urban mental diseases during the epidemic.

On the other hand, The research of crime risk assessment mainly constructs the ranking and comparison between neighbors through accessibility and surrounding environment (Newton et al., 2014; Coppola & Fasolino, 2021), which is difficult to realize the dynamic tracking and evaluation of people. In previous studies, the relationship between crime and emotion has been carefully verified (Chen et al., 2015). By detecting topics related to different emotions in different places, we can detect the correlation between these topics with different emotional polarities and specific criminal activities. Further analysis can answer the question “which area is more likely to commit crimes”. And the method of emotional Spatio-temporal analysis can dynamically track and monitor people’s psychological state in different areas of the city, and to some extent, it can solve the problem of predicting the risk period.

### Limitations

There are some limitations in this study. The Weibo data are biased, which is reflected in the age distribution of the users, and the research in this paper may ignore younger and older people. The data are not adequate in terms of spatial distribution, and the number of points for publishing Weibo in the peripheral urban areas is lower, so the representativeness is poor. Although the emotions expressed on the social media network cannot fully represent the emotions of the public in the real world, they can indeed reflect the public’s opinion to a certain extent. However, the causes of the temporal and spatial differences in emotions were not analyzed in detail in this study. In the follow-up study, we should make full use of multimodal social media data (Huang et al., 2020c) to mine emotions, and should consider social environmental factors and explore the correlation between them.

## Conclusions

In this study, an efficient multi granularity emotion classification model was constructed using pre-trained Bert and a convolution neural network, the emotional tendency of 211,526 Sina Weibo data with geographical locations in Nanchang was calculated, the spatial distribution characteristics of urban Weibo emotions were determined through emotional hotspot analysis, and the emotional topics were analyzed using word shift graphs. To further clarify the temporal and spatial variation characteristics of the emotions, the temporal and spatial patterns and trends of the Weibo emotions from day to night and from working day to the weekend were analyzed on the grid-scale. The results of this study reveal the characteristics of the temporal and spatial distributions of the Weibo emotions in the city. Compared with traditional research, Weibos have the advantages of more granular emotion types and increased temporal and spatial granularity. This provides a method for research on urban emotions and auxiliary support for analyzing the happiness of urban residents and guiding urban management and planning.

## Figures and Tables

**Fig. 9:**
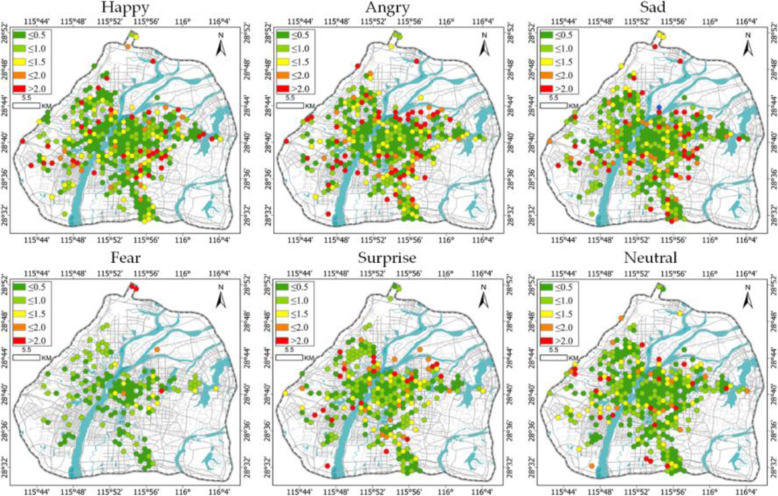
The spatial pattern of the LISA of the emotions from day to night

**Fig. 10:**
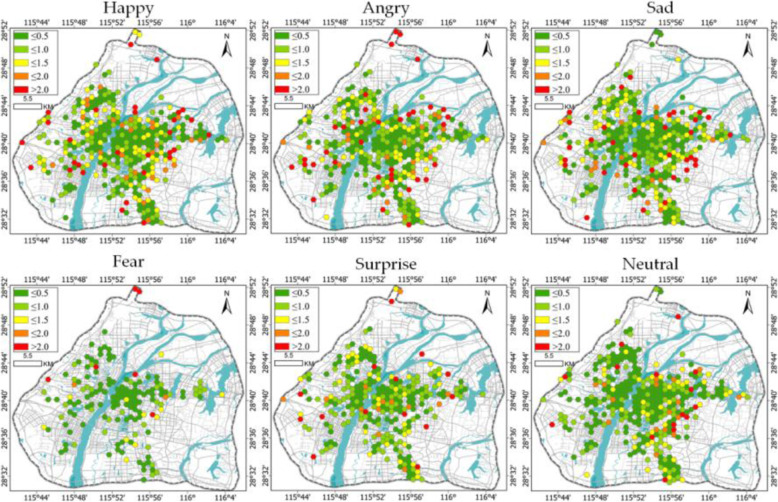
The spatial pattern of the LISA of the emotions from weekday to weekend

## Data Availability

Not applicable.
